# Editorial: Hikikomori: an international perspective on assessment, treatment, and community intervention

**DOI:** 10.3389/fpsyt.2023.1297898

**Published:** 2023-10-18

**Authors:** T. Wing Lo, John C. M. Wong, Gloria Hongyee Chan, Takahiro A. Kato

**Affiliations:** ^1^Caritas Institute of Higher Education, Tseung Kwan O, Hong Kong SAR, China; ^2^National University of Singapore, Singapore, Singapore; ^3^City University of Hong Kong, Kowloon, Hong Kong SAR, China; ^4^Kyushu University, Fukuoka, Japan

**Keywords:** hikikomori, assessment, intervention, hidden youth, social withdrawal, Japan, Singapore, Hong Kong

Hikikomori is the phenomenon of staying at home almost every day and avoiding social participation for more than 6 months (1). Hikikomori is rooted in Japan, and now spreading globally, causing a huge burden in health, welfare, education, and economy (2, 3).

There are seven publications in this Special Research Series on hikikomori, including four original research reports, two brief research reports and one community case study. All papers reported research or intervention results in East and South-east Asian regions, specifically Japan, Singapore, and Hong Kong. While the papers indicate that there are common risk factors of hikikomori, the age and gender of this target group is quite diverse.

Regarding the age of hikikomori, the papers covered different age groups, such as 12–21 years old (Chan), 12–25 years (Khiatani et al.), 20–22 years (Hihara et al.), 18–35 years (Lin et al.), 23–57 years (Yamada et al.), and 44 years (mean age, Nonaka and Sakai). This diversity indicates that the hikikomori population covers teenagers, youth, younger adults, older adults, and emerging adults (Lin et al.), suggesting that hikikomori suffering is a life-long issue if not handled properly or earlier. Those suffered also include parents or caregivers of the hikikomori (mean age: 65.6 and 67.9, in Kubo et al.) who face stress and trouble in taking care of the hikikomori.

Regarding the gender of hikikomori, the ratios between male and female as reported in the studies are: 76.5 vs. 23.5% (Chan); 21 vs. 79% (Hihara et al.); 71 vs. 29% (Nonaka and Sakai); and 43 vs. 57% (Lin et al.). These ratios suggest that the hikikomori phenomenon is not dominated by one sex.

Regarding the length of social withdrawal, most studies adopted a common definition, that is, withdrawal for at least 6 months (Chan; Hihara et al.; Kubo et al.; Nonaka and Sakai; Yamada et al.). However, one Singapore study (Lin et al.) used 3–6 months or more as the criteria, whereas another Singapore study viewed isolation between 3 and 6 months as pre-hikikomori (Khiatani et al.).

During this withdrawal period, majority of the hikikomori are involved in (a) physical withdrawal; (b) lack of social participation and interactions, such as working, attending school, and out-of-home social activities; and (c) psychological detachment, distress, depression, and anxiety as a result of social isolation (Hihara et al.; Lin et al.; Nonaka and Sakai). Singapore social workers (Khiatani et al.) also classified hikikomori with mild, moderate, severe symptoms. Those in the “mild” group leave home up to three times a week but still refrain from social interactions. Those in the “moderate” group do not leave home, yet with some family contact. Those in the “severe” group stay within their own rooms mostly without any family interaction.

Despite there is a well-recognized definition of hikikomori, a study (Nonaka and Sakai) attempted to add one more criterion: frequency of outing, to its definition. The study found that outing frequency can be considered as a condition for hikikomori, but the quality of outing, i.e., outings with or without interpersonal interaction, should also be considered. Kato et al. (1, 3) have also proposed the importance of evaluating the quality of outing in order to diagnose pathological social withdrawal condition. According to such criterion, Kato's research laboratory has just developed a structured diagnostic tool: the Hikikomori Diagnostic Evaluation (HiDE) with a structured interview and a self-rated screening questionnaire (4).

The seven papers in this Research Topic cover diversified topics: three papers are related to intervention programs, including a comparison of the use of cognitive behavioral therapy, narrative therapy and play therapy to counsel hikikomori (Chan), intervention program for family members of hikikomori (Kubo et al.), and a hidden youth intervention program in Singapore (Khiatani et al.). Another three papers address the risk factors associated with social withdrawal (Lin et al.), including job search and identity distress (Hihara et al.) and the association between autism spectrum disorder (ASD) and hikikomori (Yamada et al.).

From the Table 1, readers can find the scales used in the seven studies which on the one hand, indicate the research objectives of each study, and on the other hand, reflect the problems faced by the hikikomori, such as identity distress, life satisfaction, career expectation, internet addiction, social stigma, problematic and adaptive behavior, and solitude. The most common scales used by the studies in measuring Hikikomori symptoms of social withdrawal is the HQ-25 (5), adopted in three studies. Other symptoms commonly faced by the hikikomori are stress, anxiety, and depression. Fours studies have adopted different scales (e.g., PHQ-9) to measure these issues.

**Table 1:** Scales used by the seven studies in this special issue.

**Scales**	**Hihara et al.**	**Kubo et al.**	**Lin et al.**	**Chan**	**Nonaka and Sakai**	**Yamada et al.**	**Khiatani et al.**
Hikikomori symptoms: social withdrawal tendencies over the past 6 months	25-item Hikikomori Questionnaire (HQ-25)		HQ-25			HQ-25	Mild, moderate, severe symptoms assessed by social workers
Identity distress	Identity Distress Survey						
Life satisfaction	Satisfaction with Life Scale						
Career expectation	Self-constructed						
Perceived skills		MHFA					
Confidence		MHFA					
Stigma toward mental health problems		Japanese version of the 12-item Link's Devaluation-Discrimination Scale					
Stress/Anxiety/ Depression	Center for Epidemiological Studies Depression Scale	Japanese version of the Patient Health Questionnaire (PHQ-9) Stress Response Scale-18 (SRS-18)	Depression, Anxiety, Stress Scale–21 (DASS-21)			Patient Heath Questionnaire 9 (PHQ-9); 22-Item Tarumi's Modern-Type Depression Trait Scale (TACS-22) Liebowitz Social Anxiety Scale Japanese Version (LSAS-J); Mini Social Phobia Inventory (Mini SPIN)	
Problematic behaviors of hikikomori sufferers		Hikikomori Behavior Checklist (HBCL)					
Adaptive behaviors of hikikomori sufferers		Adaptive Behaviors Scale for Hikikomori (ABS-H)					
Attitude toward hikikomori sufferers		Family Attitude Scale (FAS)					
Hikikomori risk factors			NHR Scale				R.A.I.N. (risk, assets, interests, and needs); Risk-need-responsivity model.
Social engagement with family and friends			Lubben Social Network Scale				
Regulation of emotions			ERQ				
Psychological capital				Psychological Capital Questionnaire (PCQ-24)			
Therapy				Self-constructed items on cognitive behavioral therapy, narrative therapy, and play therapy			
Qualitative indicators of outings					3 Self-constructed items		
Subjective social functioning impairment					1 Self-constructed items		
Preference for solitude						Preference for Solitude Scale (PSS)	
Level of internet dependence						Internet Addiction Test (IAT)	
Sensory processing tendencies						Adolescent/Adult Sensory Profile (AASP)	

In 2022, hikikomori has been included in the “Culture and Psychiatric Diagnosis” Section of the DSM-5-TR, and is highlighted as a global mental health issue (6). Future research should extend the study of hikikomori to other regions of the world to explore the similarity and differences in risk factors of hikikomori under different cultural and socio-economic contexts (7). Moreover, when compared with the first special series conducted in 2018–19 at Frontiers in Psychiatry (8), researchers have paid more attention on intervention programs that addressed the needs of hikikomori and caregivers. It is expected that this research trend will continue so that more service recipients can be benefited from the programs.

## Author contributions

TL: Writing—original draft, review and editing. JW: Review. GC: Review. TK: Writing—review and editing.

## References

[1] Kato TA Kanba S Teo AR. Defining pathological social withdrawal: proposed diagnostic criteria for hikikomori. World Psychiatry. (2020) 19:116–7. 10.1002/wps.20705 31922682 PMC6953582

[2] Chan G Lo TW. The effect of negative experiences on delinquent behavior of youth in a social withdrawal situation. J Adolesc. (2016) 50:69–80. 10.1016/j.adolescence.2016.05.002 27232102

[3] Kato TA Kanba S Teo AR. Hikikomori: multidimensional understanding, assessment, and future international perspectives. Psychiatry Clin Neurosci. (2019) 73:427–40. 10.1111/pcn.12895 31148350

[4] Teo AR Horie K Kurahara K Kato TA. The Hikikomori Diagnostic Evaluation (HiDE): a proposal for a structured assessment of pathological social withdrawal. World Psychiatry. (2023) 22:478–9. 10.1002/wps.21123 37713577 PMC10503919

[5] Teo AR Chen JI Kubo H Katsuki R Sato-Kasai M Shimokawa N. Development and validation of the 25-item Hikikomori Questionnaire (HQ-25). Psychiatry Clin Neurosci. (2018) 72:780–8. 10.1111/pcn.12691 29926525 PMC6221010

[6] APA. Diagnostic and Statistical Manual of Mental Disorders, Text Revision (DSM-5-TR). Arlington, VA: American Psychiatric Press, Inc. (2022).

[7] Chan G Lo TW. Hidden youth services: what Hong Kong can learn from Japan. Child Youth Serv Rev. (2014) 42:118–26. 10.1016/j.childyouth.2014.03.021

[8] Wong JCM Wan MJS Kroneman L Kato TA Lo TW Wong PW-C. Hikikomori phenomenon in East Asia: regional perspectives, challenges and opportunities for social health agencies. Front Psychiatry. (2019) 10:512. 10.3389/fpsyt.2019.00512 31396114 PMC6663978

